# Urokinase receptor and resistance to targeted anticancer agents

**DOI:** 10.3389/fphar.2015.00154

**Published:** 2015-07-27

**Authors:** Steven L. Gonias, Jingjing Hu

**Affiliations:** Department of Pathology, School of Medicine, University of California, San Diego, San Diego, CA, USA

**Keywords:** uPAR, plasmin, fibrinolysis, epithelial-mesenchymal transition, cancer stem cell, metastasis, cellular senescence

## Abstract

The urokinase receptor (uPAR) is a GPI-anchored membrane protein, which regulates protease activity at the cell surface and, in collaboration with a system of co-receptors, triggers cell-signaling and regulates gene expression within the cell. In normal tissues, uPAR gene expression is limited; however, in cancer, uPAR is frequently over-expressed and the gene may be amplified. Hypoxia, which often develops in tumors, further increases uPAR expression by cancer cells. uPAR-initiated cell-signaling promotes cancer cell migration, invasion, metastasis, epithelial-mesenchymal transition, stem cell-like properties, survival, and release from states of dormancy. Newly emerging data suggest that the pro-survival cell-signaling activity of uPAR may allow cancer cells to “escape” from the cytotoxic effects of targeted anticancer drugs. Herein, we review the molecular properties of uPAR that are responsible for its activity in cancer cells and its ability to counteract the activity of anticancer drugs.

## Introduction

Adhesion receptors that mediate interactions between adjacent cells or with extracellular matrix (ECM) and at the same time, initiate cell-signaling include the integrins and members of the cadherin superfamily (Giancotti and Ruoslahti, 1999; Angst et al., 2001). The urokinase receptor (uPAR) is functionally similar to adhesion receptors in that it binds to the provisional ECM protein, vitronectin (Wei et al., 1994), and robustly activates cell-signaling (Blasi and Carmeliet, 2002). The second ligand for uPAR is the fibrinolysis protease, urokinase-type plasminogen activator (uPA), which like vitronectin, activates cell-signaling (Busso et al., 1994; Koshelnick et al., 1997). The signaling response elicited by uPA requires the amino-terminal region of uPA and not the uPA active site (Nguyen et al., 1998). Although the cell-signaling responses elicited by binding of either vitronectin or uPA to uPAR may be distinct, when uPAR is expressed at high levels, a composite response is observed, in which signaling factors controlled downstream of uPA and vitronectin are activated collectively (Eastman et al., 2012). From the structural standpoint, understanding the signaling activity of uPAR has been a fascinating challenge given that this 55-kDa, three-domain receptor is coupled to the cell surface only by a glycosylphosphatidylinositol anchor (Roldan et al., 1990; Ploug et al., 1991).

We now understand that the cell-signaling activity of uPAR controls many aspects of cell physiology that are pivotal in cancer progression. Clinical trial data support the hypothesis that uPAR is associated with cancer progression. In pancreatic cancer, the gene encoding uPAR may be amplified and this event substantially deteriorates prognosis (Hildenbrand et al., 2009). In astrocytic brain tumors, uPAR expression correlates with tumor grade (Yamamoto et al., 1994). Recent results suggest that uPAR may play an important role promoting cancer cell survival during cancer chemotherapy. We hypothesize that: *Developing new uPAR-targeting therapeutics may be advantageous to improve the efficacy of currently available anticancer agents*. Support for this hypothesis is found in numerous basic and translational studies that have explored molecular aspects of uPAR function. Unfortunately, efforts to develop uPAR-targeting drugs are still in a formative stage.

## Regulation of Cell Surface Plasminogen Activation

Early studies demonstrated that uPA-binding to uPAR increases the catalytic efficiency (k_*cat*_/K_*M*_) of plasminogen activation (Ellis and Danø, 1991). Plasminogen activation is further stimulated by the simultaneous binding of plasminogen to the cell surface, which is mediated by any of a number of membrane-associated proteins (Miles and Parmer, 2013). Anchoring of the single-chain or zymogen form of uPA to uPAR also accelerates its conversion to the enzymatically active, two-chain variant, in a reaction most frequently catalyzed by membrane-associated plasmin (Ellis, 1996). This positive feedback loop may generate large amounts of active plasmin at the cell surface.

Although best understood as the principal protease responsible for lysis of fibrin clots, plasmin has diverse glycoprotein substrates and thus, has been implicated in diverse activities including ECM remodeling, angiogenesis, cell migration, and cancer invasion (Mignatti and Rifkin, 1993). Key plasmin substrates, in addition to fibrin and ECM proteins, include latent transforming growth factor-β (Lyons et al., 1988) and pro-forms of matrix metalloproteases (MMPs; Murphy et al., 1999). Many plasmin activities may be facilitated by binding to cellular plasminogen receptors (Miles and Parmer, 2013).

Diverse cancer cells express high levels of uPAR (Mazar, 2008; Smith and Marshall, 2010) and also express plasminogen receptors that function in plasmin generation (Gonias et al., 2001). As cancers enlarge, they frequently outgrow their blood supply, causing hypoxia in the tumor core, which is a known inducer of uPAR expression (Graham et al., 1999; Lester et al., 2007). The effects of hypoxia on uPAR expression are mediated by hypoxia-inducible factor-1, which binds to the hypoxia-responsive element in the uPAR promoter (Krishnamachary et al., 2003). Although this is probably a compensatory response, meant to promote cell survival, once uPAR expression is induced, all the activities of uPAR described herein may be activated, including the ability of uPAR to promote plasminogen activation and potentiate tissue remodeling.

## uPAR Signaling Requires a Multiprotein Receptor Complex

To activate cell-signaling in response to uPA or vitronectin, uPAR utilizes a system of co-receptors, which are dynamically assembled to generate qualitatively differing responses (Jo et al., 2005). Formyl peptide receptor-1 (FPR1) is a G protein-coupled receptor and essential co-receptor for cell-signaling downstream of membrane-anchored uPAR (Resnati et al., 2002). FPR1 also mediates cell-signaling in response to soluble forms of uPAR (Resnati et al., 2002). In cells that lack FPR1, FPR2 may substitute to mediate uPAR signaling (de Paulis et al., 2004). Diverse integrins also have been implicated in uPAR signaling, including α_3_β_1_, α_5_β_1_; α_*v*_β_1_, α_*v*_β_5_, and β2 integrins such as Mac1 (Yebra et al., 1996; May et al., 1998; Carriero et al., 1999; Nguyen et al., 1999; Wei et al., 2001, 2005). Mechanistically, uPAR physically associates with integrins to regulate integrin activity in cell adhesion, cell migration, and in assembly of cell-signaling complexes. Src family kinases and focal adhesion kinase are instrumental upstream signaling factors for uPAR and probably associate with uPAR indirectly through integrins (Koshelnick et al., 1997; Nguyen et al., 2000).

gp130 associates with uPAR in some cell types and may control activation of the JAK1-STAT1 pathway (Koshelnick et al., 1997). Receptor tyrosine kinases (RTKs) also may be important in uPAR signaling. In vascular smooth muscle cells, platelet-derived growth factor receptor-β has been implicated (Kiyan et al., 2005). In many other cell types, the EGF receptor (EGFR) plays an important role (Liu et al., 2002; Jo et al., 2003, 2005). In cancer cells, the EGFR functions with uPAR to activate ERK1/2, which promotes tumor cell survival and release from states of dormancy (Ma et al., 2001; Liu et al., 2002). The EGFR and uPAR also cooperate to activate the mitogenic transcription factor, STAT5b (Jo et al., 2005). Other cell-signaling factors, which have received considerable attention as key downstream targets of uPAR signaling, include PI3K and Rho GTPases such as Rac1 (Blasi and Carmeliet, 2002; Smith and Marshall, 2010). There is considerable overlap between the pathways controlled by RTKs and uPAR. This redundancy may partially explain the ability of uPAR to substitute for RTKs and promote cell survival in tumors treated with RTK-targeting drugs.

## uPAR Expression and Function in Cancer

Targeting uPAR in cancer is intriguing given that in normal quiescent human tissues, uPAR expression is limited (Mazar, 2008; Smith and Marshall, 2010). Increased uPAR expression may be observed in activated non-neoplastic cells, including endothelium, smooth muscle cells, and immune system cells, especially in processes such as tissue injury or inflammation. By contrast, uPAR is highly expressed by diverse cancer cells and by non-malignant cells that infiltrate cancers (Mazar, 2008; Smith and Marshall, 2010). When expressed in malignancy, uPAR typically worsens the prognosis irrespective of whether the cell of origin is the tumor cell or the stromal cell.

In addition to pancreatic cancer, *uPAR* gene amplification is observed in breast cancer. In breast cancers that are HER2-positive, *uPAR* and *HER2* tend to be amplified in the same cells (Uhr, 2008). In colorectal cancer, uPAR expression by non-malignant stromal cells is correlated with a negative prognosis (Boonstra et al., 2014). Obviously, different mechanisms are operational when cancer progression is accelerated by tumor cell uPAR versus stromal uPAR. One possible pathway by which stromal cell uPAR may promote cancer progression is by releasing soluble uPAR, which is biologically active and may regulate cancer cell physiology (Gilder et al., 2015).

Early models attributed the ability of uPAR to promote cancer progression to its control of extracellular proteolysis at the cancer cell surface (Blasi and Carmeliet, 2002; Dano et al., 2005). However, we and others have shown that uPAR promotes cell migration by activating Rac1 and ERK1/2 (Nguyen et al., 1998; Kjoller and Hall, 2001; Ma et al., 2002). In a mouse xenograft model system, uPAR promoted metastasis exclusively by controlling cell-signaling factors such as Rac1 (Jo et al., 2009b). This result does not discount the importance of uPAR in extracellular proteolysis but instead, proves the importance of uPAR-dependent cell-signaling in cancer progression in an *in vivo* model system.

The ability of uPAR to promote cell survival is particularly relevant to its activity in cancer treatment. In cell culture model systems, uPA-binding to uPAR inhibits apoptosis by maintaining an increased level of phosphorylated ERK1/2 (Ma et al., 2001). Similarly, uPAR-initiated cell-signaling prevents anoikis *in vitro* by transcriptional activation of the anti-apoptotic BCL-2 family member, BCL-xL (Alfano et al., 2006). uPAR signaling also regulates BIM, which is a second BCL-2 family member that promotes apoptosis (Wykosky et al., 2015). Glioblastoma (GBM) cells may be driven into apoptosis by suppressing uPAR signaling, which elevates BIM.

Cellular senescence and tumor cell dormancy are important concepts in cancer therapy (Campisi and d’Adda di Fagagna, 2007; Gewirtz, 2009). In response to radiation or chemotherapy, tumors cells may enter senescence as opposed to undergoing apoptosis. Replicative arrest is characteristic of cellular senescence; however, so is sustained survival. From the standpoint of cancer treatment, senescence within a sub-population of tumor cells implies a diminished capacity for tumor growth but also, a decreased opportunity for cancer eradication. There is still debate regarding whether cellular senescence is fully irreversible in cancer. Ossowski and colleagues showed that uPAR controls entry of cancer cells into states of dormancy and release from dormancy (Yu et al., 1997; Aguirre Ghiso et al., 1999) and thereby demonstrated the capacity of uPAR to regulate checkpoints in the life cycle of a cancer cell.

**FIGURE 1:**
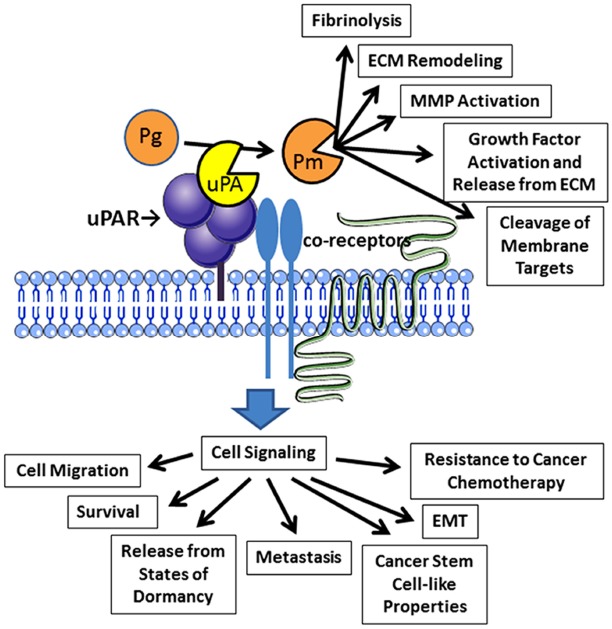
**Activities of uPAR in cancer cells.** Binding of uPA to uPAR promotes activation of plasminogen (Pg) to form plasmin (Pm). Plasmin then expresses diverse activities near the cell surface. uPAR also collaborates with a system of co-receptors to activate cell-signaling. Reported outcomes of uPAR-initiated cell-signaling in cancer cells are shown.

uPAR-initiated cell signaling promotes epithelial-mesenchymal transition (EMT) and this process appears to be reversible (Lester et al., 2007; Jo et al., 2009a). Hypoxia facilitates EMT by increasing uPAR expression (Lester et al., 2007). uPAR may play a central role in the mechanism by which gene products, such as the transcription factor, Forkhead Box M1, promote EMT (Huang et al., 2014). uPAR-activated cell-signaling also induces stem cell-like properties in cancer cells (Jo et al., 2010). Finally, uPAR controls gene expression in cancer cells, promoting expression of factors such as interleukin-4 and transforming growth factor-β, which condition immune system cells so that the tumor microenvironment is more conducive for tumor growth (Hu et al., 2014a). The activities of cancer cell uPAR are summarized in Figure 1.

## uPAR and Chemoresistance in Cancer

In studies with cell culture model systems, Alfano et al. (2006) showed that silencing uPA increases the extent of apoptosis observed when cells are treated with cisplatin or UV irradiation. In small cell lung cancer in patients, uPAR expression is associated with resistance to diverse traditional chemotherapeutic agents (Gutova et al., 2007). In squamous cell carcinoma of the head and neck and in malignant mesothelioma, uPAR confers resistance to cisplatin (Cortes-Dericks et al., 2010; Huang et al., 2013). Because uPAR appears to confer some degree of resistance to almost all forms of traditional cancer therapy, each round of treatment may select for tumor cells that have the highest uPAR expression levels. As a result, uPAR-positivity may become increasingly problematic in patients that require multiple rounds of cancer treatment with different modalities.

In breast cancers in which tumor cells express estrogen receptor, anti-estrogen therapeutics such as tamoxifen have served as effective “targeted” anticancer agents (Massarweh and Schiff, 2006). Because as many as 70% of all breast cancers are estrogen receptor-positive, understanding why some malignancies acquire resistance of anti-estrogen drugs is of considerable importance. Activation of cell-signaling pathways downstream of the EGFR, HER2, and Insulin-like Growth Factor Receptor-1 has received attention (Massarweh and Schiff, 2006). In a series of 691 breast cancer patients treated with tamoxifen, progression-free survival correlated inversely with expression of uPAR and uPA (Meijer-van Gelder et al., 2004). To study this phenomenon, we examined estrogen-dependent breast cancer cell lines (Eastman et al., 2012). In the presence of estrogen, estrogen receptor-α (ERα) functioned as a major receptor responsible for sustaining ERK1/2 activation. When estrogen was withdrawn, ERK1/2 phosphorylation decreased. To model how uPAR may regulate this process, we over-expressed uPAR in our ERα-expressing breast cancer cells. When estrogen was present, uPAR did not regulate ERK1/2 phosphorylation; however, in the absence of estrogen, uPAR provided a rescue pathway, sustaining ERK1/2 activation and promoting cell survival (Eastman et al., 2012). Similar results were obtained when we utilized a xenograft model system in mice. MCF-7 breast cancer cells typically require estrogen supplementation to establish xenografts in SCID mice; however, when MCF-7 cells were transfected to over-express uPAR, the estrogen requirement was attenuated (Eastman et al., 2012). Although more work is clearly required, these early studies support a model in which changes in uPAR expression in breast cancer cells may release tumors from control by estrogen receptor-targeting therapeutics in patients.

## uPAR and EGFR in Glioblastoma

Glioblastoma is a highly aggressive astrocytic tumor of the brain in which the gene encoding the EGFR is frequently amplified, driving tumorigenicity (Heimberger et al., 2005). In the context of *EGFR* gene amplification, *EGFR* mutations are common including a truncation mutation that generates a form of the receptor called EGF receptor variant III (EGFRvIII). This EGFR variant does not bind EGF but demonstrates constitutive enzymatic activity in the absence of growth factor (Nishikawa et al., 1994; Heimberger et al., 2005). Given the robust effects of *EGFR* gene amplification and EGFRvIII on GBM progression, it would be reasonable to assume that EGFR-targeting therapeutics would be effective in treating GBM; however, although temporary responses may be observed, tumors typically escape from control (Voelzke et al., 2008).

To understand why EGFR-targeting therapeutics do not demonstrate greater efficacy in GBM, together with our colleagues, we evaluated three models of acquired resistance to EGFR-targeting drugs. Mukasa et al. (2010) developed the first model, applying a genetic approach. EGFRvIII was expressed in U373MG GBM cells under the control of a doxycycline-repressible promoter. Tumors were developed in mice. Once the tumors were established, EGFRvIII expression was neutralized *in vivo* forcing the tumors into a state of dormancy. Many of these tumors emerged from dormancy, re-establishing growth. Wykosky et al. (2015) developed two additional model systems in which GBM cells were treated with the EGFR-targeting tyrosine kinase inhibitors (TKIs), erlotinib, and gefitinib, either in three dimensional cell culture or in xenografts *in vivo*. TKI resistance developed and was readily documented in cell viability and proliferation assays.

In all three model systems, neutralization of EGFRvIII activity induced expression of uPA, activating uPAR-dependent cell-signaling (Hu et al., 2011; Wykosky et al., 2015). uPAR assumed a major role sustaining ERK1/2 activation (Hu et al., 2011; Wykosky et al., 2015). As a result, apoptosis was prevented and the GBM cells survived. BIM was a major target for ERK1/2, downstream of uPAR in GBM cells. Silencing uPA in TKI-resistant GBM cells increased BIM levels and promoted apoptosis. Inhibiting MEK or treating cells with a BH-3 mimetic, which counteracts the activity of anti-apoptotic Bcl-2 family members, restored sensitivity to TKIs in GBM cells. These results suggest that the uPA-uPAR signaling system may provide a major escape pathway for GBM cells when tumors are treated with EGFR-targeting therapeutics. Interestingly, when EGFRvIII was neutralized in GBM cells, GBM cell migration was potentiated (Hu et al., 2014b). The compensatory response of the GBM cells, which involved activation of uPAR signaling to promote cell survival, also promoted cell migration, which is a well described consequence of uPAR-activated cell-signaling. These results suggest that changes in uPAR expression in cancer cells, induced or selected for by anticancer therapies, may unintendedly increase the capacity of the cancer cells to invade or metastasize.

## Concluding Comments

Urokinase receptor regulates activities that are relevant to cancer progression on both sides of the plasma membrane. At the cell surface, uPAR stimulates tissue remodeling. Cell-signaling pathways, activated downstream of uPAR, stimulate many activities implicated in cancer progression. As a response to conventional or targeted anticancer agents, uPAR signaling may be activated. Alternatively, anticancer drugs may select for cancer cells in which uPA or uPAR are most highly expressed. Because cell-signaling pathways that support cell survival also may promote cell migration, activation of uPAR-dependent cell-signaling in treated cancer cells may not only prevent cancer eradication but also promote cancer progression.

### Conflict of Interest Statement

The authors declare that the research was conducted in the absence of any commercial or financial relationships that could be construed as a potential conflict of interest.

## Abbreviations

ECM: extracellular matrix
EGFR: EGF receptor
EGFRvIII: EGF receptor variant III
EMT: epithelial-mesenchymal transition
ERα: estrogen receptor-α
GBM: glioblastoma
MMP: matrix metalloprotease
RTK: receptor tyrosine kinase
TKI: tyrosine kinase inhibitor
uPA: urokinase-type plasminogen activator
uPAR: urokinase receptor.
